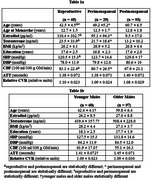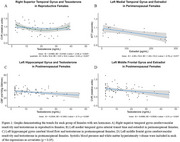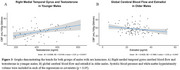# Sex Hormones and Cerebrovascular Health: Implications for Dementia Risk Across the Lifespan

**DOI:** 10.1002/alz70862_109774

**Published:** 2025-12-23

**Authors:** Brittany Intzandt, Zacharie Potvin‐Jutras, Kevin Whittingstall, Claudine J Gauthier

**Affiliations:** ^1^ Dr. Sandra E. Black Centre for Brain Resilience and Recovery, LC Campbell Cognitive Neurology, Hurvitz Brain Sciences Program, Sunnybrook Research Institute, University of Toronto, Toronto, ON Canada; ^2^ Sunnybrook Research Institute, Toronto, ON Canada; ^3^ Concordia University, Montreal, QC Canada; ^4^ Montreal Heart Institute, Montreal, QC Canada; ^5^ Centre de Recherche de L'Institut de Cardiologie de Montréal, Montreal, QC Canada; ^6^ Université de Sherbrooke, Sherbrooke, QC Canada

## Abstract

**Background:**

Circulating sex hormones decrease in aging, influencing cerebrovascular health, a key factor in dementia risk. Early menopause is a risk factor for dementia comparable to smoking^2^. Estradiol consistently supports female vasculature^1^ and brain health, though its effects on cerebrovascular health is understudied. Testosterone’s effects in males remain unclear^3,4^. Limited data exist on the sex‐ and life stage‐specific impacts of hormones on cerebrovascular health, making it critical to understand these relationships for targeting hormone‐related interventions to mitigate dementia risk

**Method:**

187 females from the Human Connectome Aging Project were categorized according to STRAW criteria as reproductive (*n* = 65), perimenopausal (*n* = 29) or postmenopausal (*n* = 93). 157 males were as younger [36 to 49 years] (*n* = 60) or older [50 to 70 years] (*n* = 97). MRI scans quantified cerebral blood flow (CBF), arterial transit time (ATT), relative cerebrovascular reactivity (CVR)^5^ and a blood draw for circulating sex hormones. ANCOVA’s tested group differences, and polynomial regressions assessed hormone relationships with CBF, ATT, and CVR.

**Result:**

In reproductive females, testosterone exhibited a U‐shaped relationship with CVR in the temporal lobe. Perimenopausal females demonstrated an inverse relationship between estradiol and ATT (shorter ATT indicates better cerebrovascular health) in frontal and temporal regions, and a positive relationship with testosterone and ATT in temporal and hippocampal regions. Postmenopausal females revealed inverse linear relationships with testosterone and CBF in frontal and temporal regions, and with CVR in frontal regions. In males, testosterone positively correlated with CBF in younger participants, while estradiol inversely correlated with CBF in older males.

**Conclusion:**

Sex hormones demonstrate life stage‐ and sex‐specific associations with cerebrovascular health including CBF, CVR, and ATT, all crucial factors in dementia pathogenesis. Both sexes had opposite directions of relationship for estradiol and testosterone. Estradiol was associated with better cerebrovascular health (shorter ATT in perimenopausal females), while testosterone showed negatives effects in females (longer ATT, lower CBF and CVR). Conversely, testosterone was associated with higher CBF in younger males and estradiol lower CBF in older males. These findings underscore the potential for hormone‐targeted strategies to support cerebrovascular health and reduce dementia risk through age and stage‐specific interventions.